# Effects of herb-partitioned moxibustion for diarrhoea-predominant irritable bowel syndrome: A protocol for systematic review and meta-analysis

**DOI:** 10.1097/MD.0000000000021817

**Published:** 2020-08-21

**Authors:** Fen Wang, Shuxing He, Jian Yan, Lianren Mai, Liangjun Yang

**Affiliations:** a Hainan Hospital of Traditional Chinese Medicine, Haikou; b Department of Gastroenterology, Tongde Hospital of Zhejiang Province, Hangzhou, China.

**Keywords:** diarrhea-predominant irritable bowel syndrome, herb-partitioned moxibustion, meta-analysis, protocol, systematic review

## Abstract

**Background:**

Diarrhea-predominant irritable bowel syndrome (IBS-D) is a common functional intestinal disease characterized by abdominal pain and diarrhea. Herb-partitioned moxibustion (HPM), a characteristic external therapy, is effective in treating IBS-D. However, no systematic review has been carried out to assess the efficacy and safety of HPM for IBS-D. The aim of this study will systematically evaluate the efficacy and safety of HPM for the treatment of patients with IBS-D.

**Methods:**

We will perform the comprehensive literature search in both English and Chinese electronic database including PubMed, Embase, Cochrane Library, Web of Science database, Medline, Chinese BioMedical Literature Database, China National Knowledge Infrastructure, Wanfang database, Chongqing VIP information, and SinoMed from their inception to July 2020. All randomized controlled trials of HPM for the treatment of IBS-D will be included. RevManV5. 3 will be applied to analyze the data.

**Results:**

This study will provide high-quality synthesis of current evidence of effectiveness and safety on HPM for patients with IBS-D.

**Conclusion:**

The conclusion of our systematic review will provide evidence to judge whether HPM is an effective intervention for IBS-D.

**Trial registration number:**

10.17605/OSF.IO/3JXCZ.

## 1 Introduction

Diarrhea-predominant irritable bowel syndrome(IBS-D), a subtype of IBS, is one of the most common functional bowel disorders in which people experience frequent episodes of diarrhea accompanied by abdominal pain.^[1]^ Currently, it is estimated that IBS has a prevalence of approximately 6.5% to 10% in the Asian population, and one-third of whom have IBS associated with diarrhea.^[2,3]^ This disorder has a marked negative impact on the quality of life of the patient and consumes a large amount of limited medical resources.^[4]^ The current drugs for IBS-D include antispasmodics, anti-motility drugs, and anti-depressants.^[5]^ Although these medications play a vital role in the treatment of IBS-D, the main effect is only to temporarily relieve symptoms, and these symptoms have a high relapse rate.^[6]^ Therefore, it is of great significance to seek new therapies for the treatment of IBS-D.

In recent years, traditional Chinese medicine (TCM) has attracted extensive attention for its ability to treat gastrointestinal diseases due to its moderate treatment effect and lower side effect. Herb-partitioned moxibustion (HPM), a TCM therapy suitable for some chronic diseases, has been used to treat primary dysmenorrhea, Crohn disease, and IBS-D.^[7–9]^ Clinical study has demonstrated that HPM has a better effect on ameliorating the symptoms than patients orally taking pinaverium bromide.^[9]^ Moreover, an experiment on the rat model of IBS-D indicated that HPM had beneficial effects by re-normalizing IBS-induced metabolic changes.^[10]^ Although many studies have shown that HPM is effective for patients with IBS-D. No systematic review has been carried out to assess the efficacy and safety of HPM for IBS-D. Thus, this study will systematically evaluate the efficacy and safety of HPM for the treatment of patients with IBS-D.

## 2 Objectives

The aim of this study is designed to perform a systematic assessment of the effectiveness and safety of HPM for the treatment of IBS-D.

## 3 Material and methods

This systematic review and meta-analysis protocol has been registered on Open Science Framework (OSF, https://osf.io/). The registration DOI of this study is 10.17605/OSF.IO/3JXCZ. The procedure of this protocol will be conducted following the preferred reporting items for systematic reviews and meta-analysis (PRISMA) statement.^[11]^

### 3.1 Inclusion and exclusion criteria

#### 3.1.1 Types of studies

This work will include all randomized controlled trials (RCTs) that evaluate the efficacy and safety of HPM on patients with IBS-D without any language or date of dissemination or publication status restrictions. Non-RCTs, observational studies, animal experiments, reviews, and case reports will be excluded.

#### 3.1.2 Types of participants

The target population is people with a confirmed clinical diagnosis of IBS-D according to the Rome II, III, or VI criteria without considering any information related to their age, gender, race, education, nationality, or economic status.

#### 3.1.3 Types of interventions

The experimental group will receive HPM therapy or combined with routine treatment recommended by guidelines without limitation to the intervention duration and frequency. Control interventions will include no treatment, placebo, and routine pharmacotherapies.

#### 3.1.4 Types of outcome measures

The following primary outcomes will be measured: average weekly stool frequency, visual analog scale (VAS), and the Bristol scale. The secondary outcomes mainly include the gastrointestinal symptom rating scale (GSRS), SF-36, IBS-QOL, IBS-SSS, rectal perception, and adverse events.

### 3.2 Search strategy for the identification of studies

We will perform the comprehensive literature search in both English and Chinese electronic database including PubMed, Embase, Cochrane Library, Web of Science database, Medline, Chinese BioMedical Literature Database, China National Knowledge Infrastructure, Wanfang database, Chongqing VIP information, and SinoMed from their inception to July 2020. The search strategy in Pubmed is as follows:

1. Search: (((((moxibustion [mesh terms])) or (herb partitioned moxibustion [title/abstract])) or (herb partitioned [title/abstract])) or (herbal cake-partitioned moxibustion [title/abstract]).
2. Search: ((((irritable bowel syndrome [mesh terms]) or (IBS [title/abstract])) or (diarrhea [mesh terms])) or (IBS-D [title/abstract])) or (diarrhoea predominant irritable bowel syndrome [title/abstract]).
3. Search ((((((((randomized controlled trials [mesh terms]) or RCT [title/abstract]) or controlled clinical trial [mesh terms]) or randomized [title/abstract]) or randomly [title/abstract]) or random [title/abstract]) or controlled [title/abstract]) or control [title/abstract]) or trial [title/abstract].
4. #1 and #2 and #3

### 3.3 Data collection and analysis

#### 3.3.1 Selection of studies

**Figure 1:**
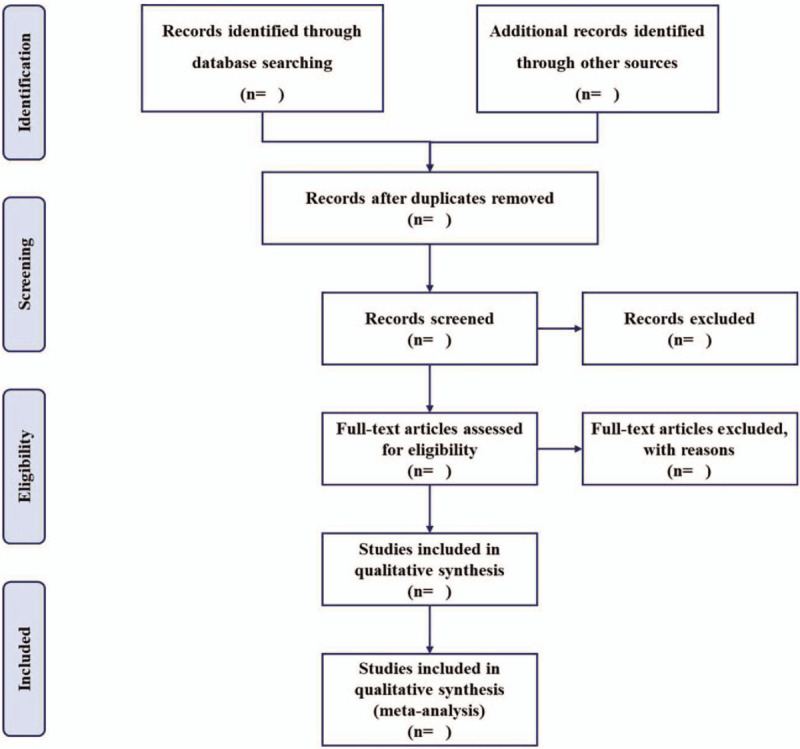
Flow chart of study selection.

According to the research criteria and search strategies, 2 reviewers will independently identify all relevant studies and sequentially screen their titles, abstracts, and keywords for eligibility after removing duplications. The articles that meet the criteria will be further determined for inclusion by reviewing the full-text. Any disagreements will be resolved through discussion to get a consensus. A PRISMA flow chart will be produced to show the number of articles identified, screened, included, and excluded (shown in Fig. 1).

#### 3.3.2 Data extraction and management

Two independent reviewers will extract data from included studies. The characteristic information including the year of study publication, sample size, age, dropouts, study period, intervention details, outcomes, and adverse events will be extracted. If needed, the corresponding authors of the primary studies will be contacted.

#### 3.3.3 Assessment of risk of bias in included studies

Two reviewers will assess the risk of bias of included articles by using the Cochrane collaboration tool. In this tool, the risk of bias of a trial will be assessed through 7 items: random sequence generation, allocation concealment, blinding of the participants and personnel, blinding of the outcome assessments, incomplete outcome data, selective reporting, and other bias. Each item will be classified as “low risk”, “high risk” or “unclear risk”. Any disagreements will be solved by a discussion of all reviewers

#### 3.3.4 Measures of treatment effect

For continuous data, the mean difference (MD) or standardized MD with 95% confidence interval (CI) will be calculated. For dichotomous outcomes, we will calculate the date and present it by the relative risk (RR) with 95% CI.

#### 3.3.5 Dealing with missing data

If data information is missing in the included studies, we will contact the corresponding author of articles by email to request missing data. If it fails, we will analyze it based on available data. Secondly, we will discuss the impact of missing data and analyze the potential impact on the results of this review.

#### 3.3.6 Assessment of heterogeneity

The heterogeneity will be analyzed through Chi-squared (*X*^*2*^) test and *I*^*2*^ tests according to the Cochrane Handbook.^[12]^ When *P*≥.05 and *I*^2^≤50%, it is considered that there is no statistical heterogeneity or the heterogeneity is small between these studies. When *P*<.05 and *I*^*2*^>50%, the study will be considered to have substantial heterogeneity.

#### 3.3.7 Data synthesis and analysis

The meta-analysis will be performed by using RevMan V5.3 (the Nordic Cochrane Centre, Copenhagen, Denmark). If there is no statistic heterogeneity, the fixed effects model will be used for analysis. If there is significant heterogeneity, a random-effects model will be used for meta-analysis. If obvious clinical heterogeneity is observed in this review, further subgroup analysis or sensitivity analysis will be performed.

#### 3.3.8 Assessment of reporting bias

If there are more than 10 trials included in the study, a funnel plot will be used to judge whether there is a publication bias.

#### 3.3.9 Subgroup analysis

Subgroup analysis according to the different acupoints, durations of treatment, and outcome measures will be performed to find the source of heterogeneity.

#### 3.3.10 Sensitivity analysis

To verify the stability of the outcomes, sensitivity analysis will be conducted to determine the robustness of the results by ruling out studies of low quality and small sample size.

#### 3.3.11 Grading the quality of evidence

The grading of recommendations assessment, development, and evaluation (GRADE) will be applied to evaluate the quality of evidence.^[13]^ The quality of evidence will be classified into “very low”, “low”, “moderate”, or “high” judgment.

#### 3.3.12 Ethics and dissemination

There is no need for a requirement of ethical approval and informed consent for this study because it is based on published literature. The results of this work will be published in a peer-reviewed journal.

## 4 Discussion

As one of the most common gastrointestinal disorders, IBS-D is highly prevalent throughout the world and is characterized by abdominal pain and altered bowel habits. Numerous studies in China have provided good evidence showing that HPM is efficient in the treatment of IBS-D. Currently, to the best of our knowledge, there is no systematic review related to HPM for IBS-D. Therefore, we conduct this systematic review to further study the effectiveness of HPM in treating IBS-D. The results of this review will provide clinicians with information about the credibility of current evidence and research direction in the treatment of IBS-D.

## 5 Amendments

If amendments are needed, we will update our protocol to include any changes in the whole process of research.

## Author contributions

**Conceptualization:** Fen Wang, Liangjun Yang.

**Data curation:** Fen Wang, Shuxing He, Jian Yan, Lianren Mai, Liangjun Yang.

**Formal analysis:** Fen Wang, Shuxing He.

**Funding acquisition:** Jian Yan
